# Few-Femtosecond C_2_H_4_^+^ Internal Relaxation Dynamics Accessed by Selective Excitation

**DOI:** 10.1021/acs.jpclett.2c02763

**Published:** 2022-11-29

**Authors:** Matteo Lucchini, Benoit Mignolet, Mario Murari, Cayo E. M. Gonçalves, Giacinto D. Lucarelli, Fabio Frassetto, Luca Poletto, Françoise Remacle, Mauro Nisoli

**Affiliations:** † Department of Physics, Politecnico di Milano, 20133 Milano, Italy; ‡ Institute for Photonics and Nanotechnologies, IFN-CNR, 20133 Milano, Italy; ¶ Theoretical Physical Chemistry, UR MOLSYS, University of Liège, B4000 Liège, Belgium; § Institute for Photonics and Nanotechnologies, IFN-CNR, 35131 Padova, Italy

## Abstract

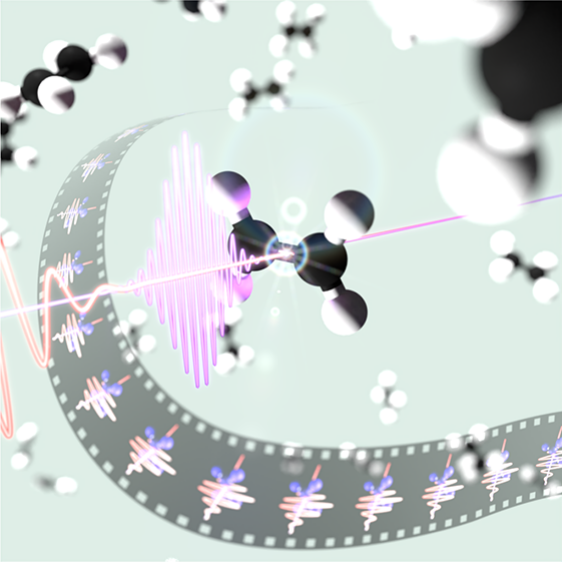

Dissociation of the ethylene cation is a prototypical multistep pathway in which the exact mechanisms leading to internal energy conversions are not fully known. For example, it is still unclear how the energy is exactly redistributed among the internal modes and which step is rate-determining. Here we use few-femtosecond extreme-ultraviolet pulses of tunable energy to excite a different superposition of the four lowest states of C_2_H_4_^+^ and probe the subsequent fast relaxation with a short infrared pulse. Our results demonstrate that the infrared pulse photoexcites the cationic ground state (GS) to higher excited states, producing a hot GS upon relaxation, which enhances the fragmentation yield. As the photoexcitation probability of the GS strongly depends on the molecular geometry, the probing by the IR pulse provides information about the ultrafast excited-state dynamics and the type of conical intersection (planar or twisted) involved in the first 20 fs of the nonradiative relaxation.

The photochemistry of many molecular systems is defined by the ultrafast dynamics which unfold in the first femtoseconds after optical excitation.^1^ The investigation of these dynamics is not trivial, both experimentally and theoretically. From the experimental point of view, it requires few-femtosecond light pulses.^2,3^ Theoretically, the full brute-force description of a many-body system becomes impractical already at the atomic level, when more than two electrons are present. As a result, most of the ultrafast dynamics initiated by light pulses remain unexplored despite their importance. A remarkable example is the relaxation dynamics of the simplest π-radical system:^4^ the ethylene cation C_2_H_4_^+^. Its dissociation is a typical multistep pathway^5−7^ in which the exact mechanisms leading to internal energy conversions are not fully known^8−10^ and recent theoretical investigations proposed different mechanisms which are not completely compatible with one another.^8,11^ For this reason, it is still unclear how the energy is redistributed among the internal modes and which step is rate-determining. In this Letter we have used 15 fs EUV femtosecond pulses in combination with short (15 fs) and intense infrared (IR) pulses to study the ultrafast molecular relaxation in a pump–probe scheme. EUV pulses of tunable photon energy are used to create a coherent superposition of the four lowest C_2_H_4_^+^ states and initiate relaxation dynamics which eventually lead to molecular fragmentation. The IR pulse further photoexcites the molecule leading to the formation of a hot ground state,^12^ which then enhances the fragmentation yield. The photoexcitation strongly depends on the initial states accessed by the EUV pulse and on the EUV-IR pulse delay. Excited-state dynamical simulations with surface hopping^13^ (SH) allowed us to identify the IR excitation mechanism as a 3-photon absorption process which takes place mainly on the cationic ground state, D0, and maximizes only for particular molecular geometries (see Figure 1a). This proves the dynamics observed in the final fragmentation yield to be sensitive to the actual population on D0. As a consequence, the timing of the C_2_H_4_^+^ pump–probe signal directly interviews the overall relaxation process, shedding new light onto the role of subsequent IR excitation and opening new routes toward the manipulation and control of complex relaxation processes in organic molecules with few-femtosecond pulses.

**Figure 1:**
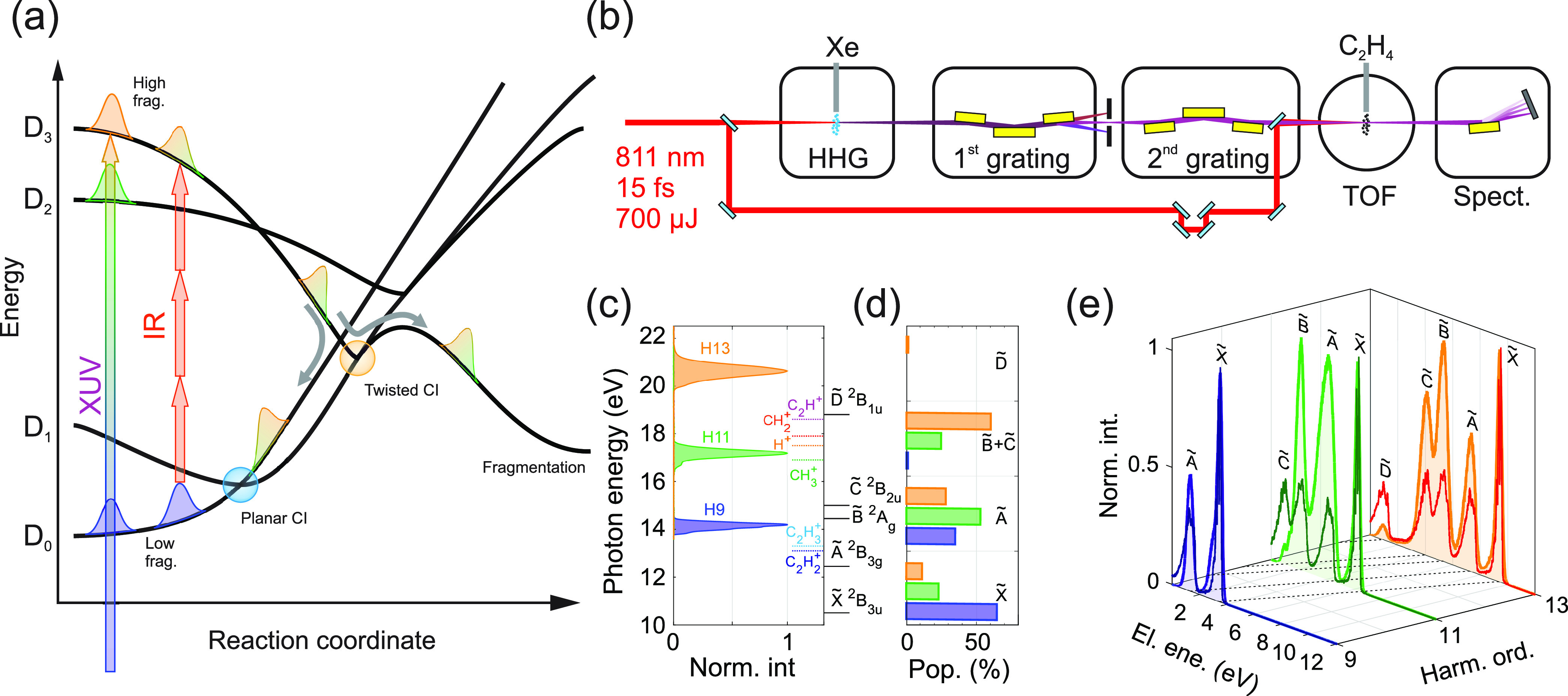
(a) Relaxation scheme: the EUV radiation projects the wave packet onto the four lowest cationic states. The population of the excited states quickly relaxes to the ground state through planar and twisted conical intersections, ultimately leading to fragmentation. The higher the initial excitation, the more fragments are produced (see Figure 2a). Once relaxed onto the ground state, the molecule can be re-excited by the IR field via three-photon absorption, thus triggering further relaxation and fragmentation. (b) Schematic of the experimental setup composed by the EUV source (high-order harmonic generation, HHG), a time-delay compensated monochromator (TDCM), a TOF spectrometer, and an XUV spectrometer. (c) Spectrum of the three harmonics together with the vertical transition energies (black horizontal lines) and the fragment appearance potentials (colored dotted lines).^16^ (d) Initial state populations calculated with the partial cross sections taken from ref (4) and the harmonic spectra in panel c. (e) Photoelectron spectra obtained by ionizing C_2_H_4_ with the harmonics in panel c (same color code). The thinner curves (darker colors) represent the high-resolution spectrum taken at 40 eV from,^17^ rigidly shifted to match the actual harmonic energy.

Figure 1b reports a scheme of the experimental setup. IR pulses with a duration of 15 fs, central wavelength of about 811 nm, and peak intensity *I*_IR_ = 3.3 × 10^12^ W/cm^2^ are focused onto a Xe gas target to obtain EUV light through high-order harmonic generation (HHG).^2^ The harmonic radiation is then sent into a time-delay compensated monochromator (TDCM),^14^ which consists of two sections working in a subtractive configuration to compensate for the temporal and spectral dispersion. As a result, it is possible to select a single harmonic while preserving its original temporal characteristics.^15^ After selection, the EUV radiation is focused on an ethylene gas target placed in the focal spot of a time-of-flight (TOF) mass spectrometer. An EUV spectrometer at the end of the setup is used to inspect the harmonic spectra.

Here we used the TDCM to select the 9th, 11th, and the 13th harmonic of the fundamental (hereafter H9, H11, and H13) whose spectra are reported in Figure 1c. The time duration of each harmonic has been characterized by photoelecron cross-correlation measurements^18^ (see the Supporting Information). We found H9 to last for 15 ± 2.2 fs, while H11 and H13 have a duration of 11 ± 1.8 fs and 7.7 ± 2.4 fs, respectively. The increasing harmonic duration with decreasing photon energy is a result of the increasing residual geometrical dispersion that cannot be compensated by the TDCM.^15,19^

Since the three harmonics have photon energy comparable with the vertical transitions from the C_2_H_4_ ground state to the lowest excited states of the molecular cation (black horizontal marks in Figure 1c), it is possible to control the initial superposition of molecular states after ionization by changing the selected harmonic. Figure 1d shows the expected initial state population evaluated by multiplying the experimental cross section reported in ref (4) with the harmonic spectra of Figure 1c. As it is possible to notice, H9 is expected to enable a very efficient excitation of the cationic ground state X̃, H11 of the first excited state Ã, while H13 predominantly excites the B̃ and C̃ states. Figure 1e shows the photoelectron spectra associated with C_2_H_4_ ionization by the three harmonics. They are characterized by a peaked structure which changes with the harmonic order. To identify the origin of the peaks, we compare the experimental spectra (light colors) with the high-resolution photoelectron spectrum reported by Holland et al.^17^ (darker colors). While a quantitative agreement is not expected, the comparison allows for the identification of the excited-state signatures, thus confirming that different harmonics prepare the molecular cation in a different superposition of the lowest excited states. The reduced collection efficiency around small kinetic energies of the TOF used in the present experimental setup prevents an absolute calibration of the electron counts. Nevertheless, the results of Figure 1e qualitatively agree with the estimation of the initial populations derived by the partial cross sections (Figure 1d), suggesting that H9 can populate only the cation GS and the first excited state, while B̃ and C̃ are more efficiently populated by H11 and H13.

As the internal relaxation process following photoexcitation by a selected harmonic can eventually lead to molecular fragmentation, a complementary approach is to study the positive fragments which are created after ionization. Figure 2a shows the main ions produced with the EUV pulses (the complete fragmentation spectra are reported in the Supporting Information). As a consequence of the different spectral properties of the ionizing radiation, the fragment yields pertaining to different harmonics qualitatively differ from each other. In particular, ionization by H9 presents a clear reduced production of the C_2_H_3_^+^ and C_2_H_2_^+^ fragments, which are related to the process of H and H_2_ loss, respectively. Furthermore, in contrast to what happens with H11 and H13, there is a clear suppression of H loss, with the H_2_ loss process becoming favored (Figure 2b). An increasing H/H_2_ loss in ethylene cation with increasing excitation energy has already been observed and discussed in the literature, yet its exact origin remains unclear, and substantially different explanations have been proposed.^8,11^ To investigate the underling relaxation dynamics we followed a pump–probe scheme and further excite the molecule with the IR pulse. Figure 2c–e shows the differential ion yield, defined as the difference between the ion yield obtained with and without the IR pulse divided by the latter: *ΔY*(τ) = (*Y*_IR_(τ) – *Y*_0_)/*Y*_0_, for the first three heaviest fragments and as a function of the delay, τ, between the EUV and IR pulses. Within the accessible range we found the observed features to be largely independent of the IR probe intensity (see the Supporting Information).

**Figure 2:**
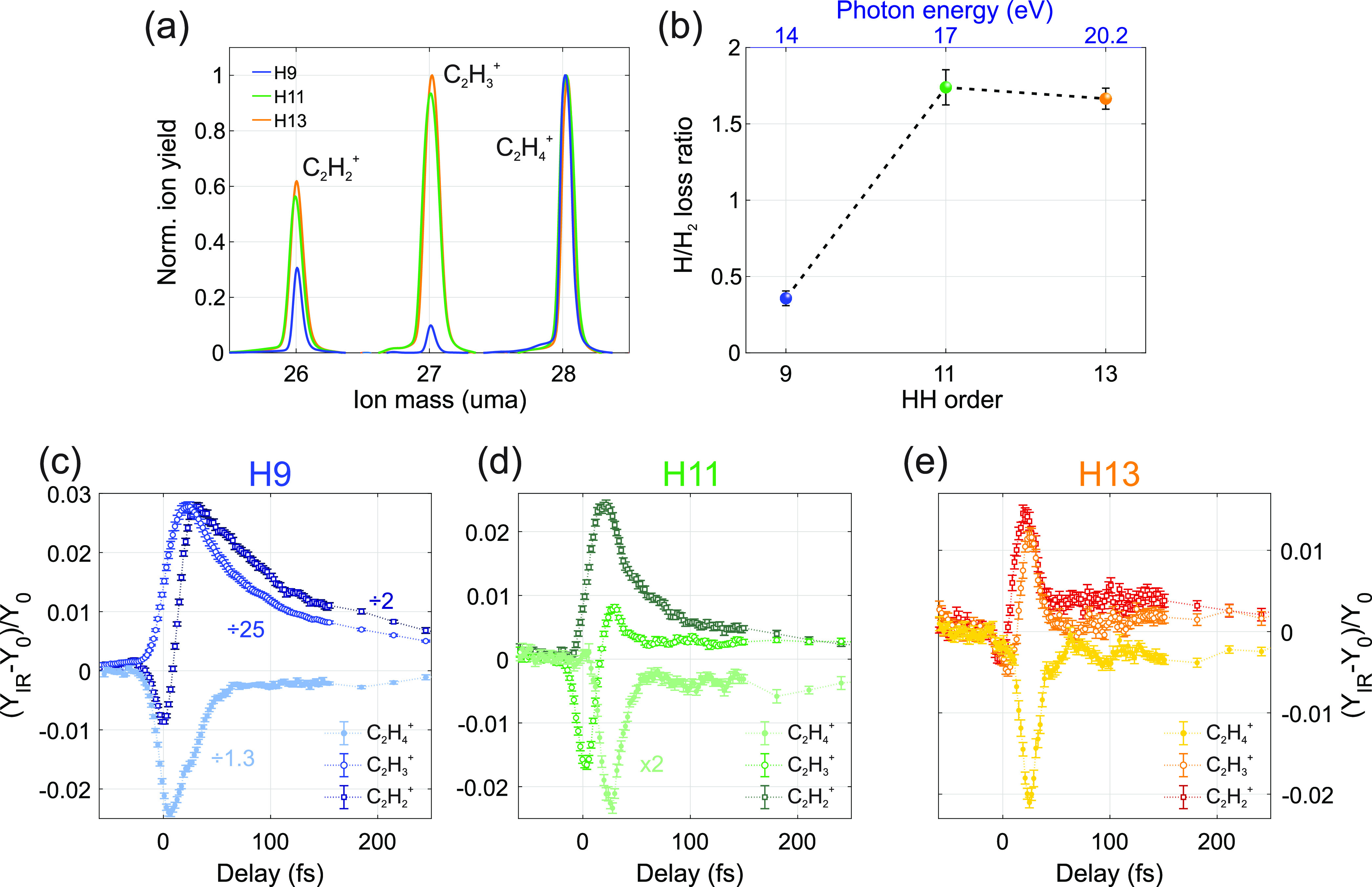
(a) Heavy fragment yields obtained with H9 (blue), H11 (green), and H13 (orange). While H13 and H11 give comparable fragment yields, H9 differs significantly. (b) H/H_2_ loss ratio after excitation with the three different harmonics. (c) Differential ion yields as a function of the delay between the XUV and IR pulse as obtained with H9. Full dots represent the C_2_H_4_^+^ yield. The C_2_H_3_^+^ and C_2_H_2_^+^ yields are displayed with open circles and squares, respectively. (d and e) Same as panel c, but for ionization with H11 and H13, respectively.

In all figures, the markers represent the average over 10 independent pump–probe measurements, while the error bars cover twice their standard deviation. Within a single scan, a mechanical shutter allows acquiring an EUV-only reference signal each two laser shots for efficient noise removal. Before and after each set of scans we perform a photoelectron pump–probe experiment which has a twofold purpose: (*i*) to extract the EUV-IR cross-correlation signal and monitor the temporal characteristics of the pulses (see the Supporting Information) and (*ii*) to precisely identify the temporal overlap between the pulses and eventually monitor any mechanical drift of the pump–probe interferometer.

The results obtained with H13 (Figure 2e) and H11 (Figure 2d) are qualitatively similar. At the pump–probe overlap the additional energy deposited by the IR pulse favors the production of small fragments (see the Supporting Information), partially bleaching the C_2_H_3_^+^ and C_2_H_2_^+^ yield (open circles and squares), but without any evident effect on the molecular cation signal (full circles). In the very first femtoseconds, the main effect of the interaction with the IR pulse by the molecular excited states, prior to internal relaxation, is thus to lead to an increased production of lighter fragments. At a delay of about 20 fs, instead, the molecular cation signal reduces and correlates to further H and H_2_ losses, which increase the C_2_H_3_^+^ and C_2_H_2_^+^ yield. The results obtained with H9 are qualitatively different (Figure 2c). The C_2_H_4_^+^ bleaching is now broader and maximizes directly at the pump probe overlap. The C_2_H_3_^+^ and C_2_H_2_^+^ relative yield exhibits a considerably slower and stronger variation. This latter could be explained as follows: ionization by an H9 photon leads to a lower production of C_2_H_3_^+^ and C_2_H_2_^+^ if compared to H11 and H13 photons (see Figure 2a,b). Nevertheless, for the molecule ionized with the H9 harmonic the absorption of several IR photons provides extra energy, which may enhance the fragmentation yield.

In the following we will concentrate on the different timing of the bleaching of the molecular cation. The belated bleaching of the C_2_H_4_^+^ signal has already been observed by Ludwig and co-workers^9^ who used a broad and energetic harmonic spectrum to ionize the molecule. For this reason they could identify neither the exact mechanism which leads to the observed delay nor the molecular state/geometry at which the interaction with the IR pulse can lead to H and H_2_ loss. Here, by combining the excitation with the different harmonics and surface hopping calculations, we find an answer to these questions.

Figure 3a–c reports the experimental molecular cation differential yields together with the result of a fit based on exponentially modified Gaussians (EMGs) (solid line). The width of the EMGs is fixed to be equal to the experimental time resolution, determined by photoelectron cross-correlation experiments (see the Supporting Information), while the amplitude, zero position, and decay rate of the EMGs are free parameters. In the case of H9 (Figure 3a), the cation reaches the minimum yield around the pump–probe temporal overlap. A second local minimum can be seen at a delay of about 20 fs. The best fit is obtained by considering the sum of a double EMG centered at τ = 0 fs (cyan dotted curve in Figure 3a) and a scaled replica, delayed by 22.4 ± 1.9 fs (violet dotted curve in Figure 3a). Each double EMG is characterized by two decay rates (a fast and a slow one), which are identical in the cyan and violet signals. The first double EMG term (cyan curve) accounts for almost 74% of the signal, while the second (violet curve) accounts for the remaining 26%. The results obtained with H11 and H13 (panels b and c of Figure 3, respectively) can instead be fitted with a single double EMG composed by a fast and slow decay. In this case, a sinusoidal component has been added to the slow EMG in order to reproduce the oscillations which can be seen at positive delays in the experimental data. The fitting procedure reveals that the oscillation period is about 50 fs, suggesting a relevant role of the torsional motion when the initial excitation happens on the higher cationic states.^8^ The bleaching of the molecular cation is centered at 19.8 ± 2.7 fs for H11 and at 22.6 ± 1 fs for H13, as observed with the weaker component for H9 (violet curve). The missing component at 0 fs for the case of H11 and H13 suggests that the C_2_H_4_^+^ bleaching is related to the nonradiative molecular relaxation. To investigate this, we first calculated the nonradiative relaxation which follows initial excitation on a single cationic state (D1 to D3) by launching 300 individual trajectories (see the Supporting Information). To account for the fact that each harmonic creates a different initial superposition of the cationic states, we then performed a weighted sum using as weights the initial populations predicted by the partial cross sections and reported in Figure 1d. The final results are reported in Figure 3d–f by the thicker curves. The thinner curves are obtained by convoluting the calculated populations with the experimental time resolution associated with each harmonic, proving it to be enough to correctly follow the relaxation process. The vertical dashed red line marks the time at which the population on the ground state D0 exceeds that of the other states. For H9 excitation (Figure 3d) this condition is matched already at *t* = 0 fs. As expected, the higher the initial photon energy, the longer it takes for the molecule to relax to D0. Nevertheless, most of the population has relaxed on D0 within 13 fs for H11 (Figure 3e) and 23 fs for H13 (Figure 3f). This confirms that the internal relaxation proceeds through ultrafast mechanisms^10,20^ and suggests a strong link to the belated bleaching observed in the experimental C_2_H_4_^+^ differential yield.

**Figure 3:**
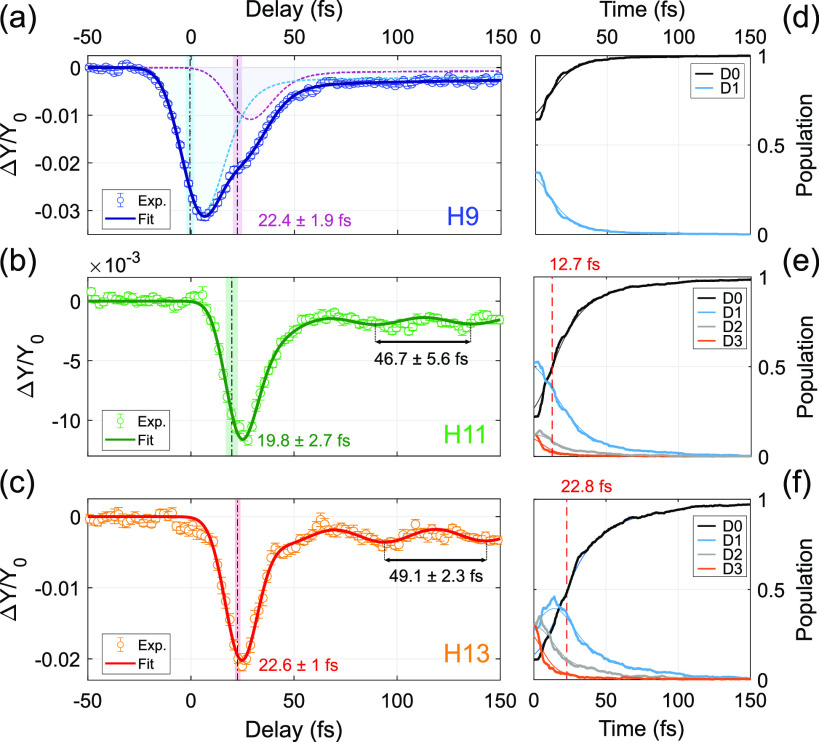
(a) Differential C_2_H_4_^+^ yield after ionization with H9 (blue markers). The solid blue curve represents the result of a fitting procedure which accounts for two delayed components (highlighted by the cyan and violet dashed curves; see related discussion in the main text). (b and c), Same quantity obtained with H11 and H13, respectively. In this case a good fit of the data is achieved with a single component which includes an oscillatory term. (d–f) Average of the simulated nonradiative relaxation of a wavepacket relaxing from the cationic states D0 to D3. The weights of the dynamics on D0, D1, D2, and D3 are given by the photoionization probability of the harmonics H9 (d), H11 (e), and H13 (f) taken from Figure 1d. The thinner lines are obtained by convoluting the calculated populations with the finite experimental time resolution.

In order to further investigate the exact nature of the ultrafast relaxation we simulated dissociation yield up to 2 ps. The results, reported in Table 1, show a strong dependence on the initial excited state. The higher the initial state, the more kinetic energy the molecular wavepacket will have after relaxation and so the higher will be the dissociation yield (0% for D0, 4% for D1, 57% for D2, and 69% for D3). In particular, the states D2 and D3 lie 4–6 eV above D0 so that upon relaxation, the molecular wavepacket has enough energy to overcome the barrier for H dissociation, which is about 2.7 eV^8^ above the mean D0–D1 energy gap. This both explains the increased C_2_H_3_^+^ and C_2_H_2_^+^ production in the static spectra of Figure 2a and offers an explanation for the pump–probe data of Figure 3. After ionization with H9, the molecule is mostly left on the cation ground state (Figure 3d); subsequent IR excitation can induce H and H_2_ loss,^21^ promptly bleaching the molecular cation signal at pump–probe overlap. With H11 and H13 the molecule is instead projected onto the excited states from which it takes a finite time to relax to D0 (Figure 3e,f) where the H and H_2_ loss can be initiated by the IR pulse. For this reason, the C_2_H_4_^+^ bleaching is delayed with respect to H9. The belated weak component observed in Figure 3a at ∼22 fs originates from the fraction of excited states which are populated by H9. Indeed, the ratio between the stronger and weaker component in the fit of the experimental C_2_H_4_^+^ yield is 74:26 and qualitatively agrees with the 65:35 ratio which describes the proportion between the initial population on D0 and on D1–D3, as extracted from the partial cross sections^4^ for the case of H9 excitation.

**Table 1:** Simulated Dissociation Yield after 2 ps of Dynamics Starting from the States D0 to D3

	dissociation yield		
initial state	C_2_H_4_^+^	C_2_H_3_^+^	C_2_H_2_^+^
D0	100%	0%	0%
D1	96%	4%	1%
D2	43%	41%	16%
D3	31%	55%	13%

Once the molecule has relaxed on D0, due to the energy spacing between the states, the interaction with the IR field can lead to resonant excitation to both D2 and D3 by three-photon absorption. Nevertheless, we found that the IR excitation is dominated by the transition D0 → D3, which is the only one displaying both a large portion of resonant wavepacket and transition dipole in the few femtoseconds after excitation (Figure 4a, see the Supporting Information).

**Figure 4:**
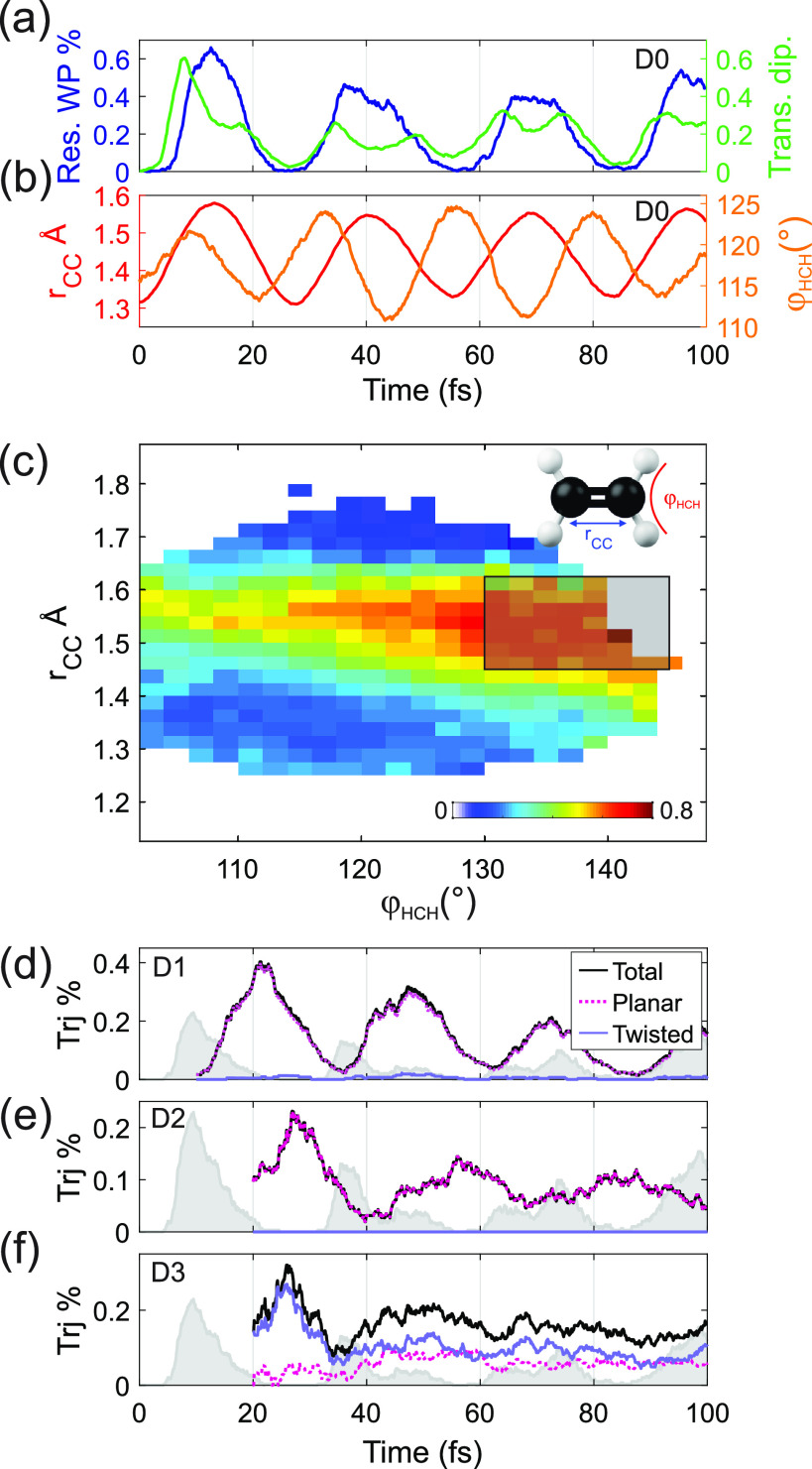
(a) Fraction of trajectories that are 3-IR-photon resonant between D0 and D3 (blue curve), together with the time evolution of the mean transition dipole moment between the same states (green curve). (b) Associated mean *r*_CC_ (red curve) and φ_HCH_ (orange curve). (c) D0–D3 transition dipole probability as a function of *r*_CC_ and φ_HCH_. The black rectangle delimits the region where the transition probability is maximized (definition in the text). (d–f) Percentage of trajectories falling into the black rectangle in panel c (solid black), calculated for initial excitation on D1, D2, and D3, respectively. The dashed magenta curve indicates the fraction of trajectories that have relaxed through a D0/D1 planar CI, while in solid violet through a twisted D0/D1 CI. The gray area indicates the time evolution on D0.

Moreover, the simulations on D0 show that the D0–D3 transition dipole moment is the largest when the CC bond, *r*_CC_, is elongated and the HCH angle, φ_HCH_, is opened (see Figure 4c). This explains why in the first 10 fs after excitation, when both *r*_CC_ and φ_HCH_ simultaneously increase (Figure 4b), the D0–D3 transition dipole moment is the largest and more than 60% of the wavepacket is 3-photon resonant. It should be noted that the D0–D2 transition dipole moment also depends on the CC elongation, but it is almost twice lower than the one for the transition D0–D3 in the first femtoseconds, indicating that the main photoexcited state is D3 (see the Supporting Information). For pump–probe time delays longer than 50 fs, there is no clear variation of the experimental C_2_H_4_^+^ yield, which could indicate that the wavepacket has spread out or that exciting D2 or D3 outside the Franck–Condon region does not increase the dissociation yield.

Maximum excitation probability to D3 is observed when both the φ_HCH_ angle is open and *r*_CC_ is elongated. To study which dynamics bring the molecule in this configuration we define a region of maximum excitation probability for 1.45 Å ≤ *r*_CC_ ≤ 1.625 Å and 130° ≤ φ_HCH_ ≤ 145° (shaded gray rectangle in Figure 4c). We note that while the transition dipole of Figure 4c stays relatively high also for angles below 130°, an extension of the defined high-excitation region toward lower values of φ_HCH_ only increases the total excitation probability without changing the associated physical interpretation (see the Supporting Information). Panels d–f of Figure 4 show the fraction of trajectories (black curve) that visits this particular geometry for different times after initial excitation on D1, D2, and D3, respectively. The nonradiative relaxation of the ethylene cation has been shown to occur either through a planar or twisted conical intersection (CI) between D0 and D1,^8^ which is represented in Figure 4d–f by the dashed magenta (planar) and the solid violet (twisted) curves. The gray area shows the percentage of trajectories associated with the maximum excitation probability for initial excitation on D0. We observe that the higher the initial excitation, the later the molecule reaches the geometry where it can be re-excited to D3, increasing fragmentation and thus decreasing the C_2_H_4_^+^ yield. When compared to initial excitation on D0, excitation on D1 leads to a maximum number of trajectories in the correct geometry about 12 fs later, while for initial excitation on D2 and D3 this configuration is reached with a delay of about 18 fs. This is consistent with the data reported in Figure 3, where the excitation with H11 and H13 produces a delayed C_2_H_4_^+^ bleaching of about 20 fs. Moreover, the calculations allow disclosing the role of planar and twisted CIs in the ultrafast relaxation dynamics of the molecular cation. Indeed, while the wavepacket excited on D1 and D2 relaxes to the right geometry almost entirely through a planar CI (Figure 4d,e), excitation to D3 (Figure 4f) relaxes both to planar and twisted CIs (almost equally divided between the two classes of CIs, in agreement with what was observed in ref (8)), but only the trajectories coming from the twisted CI can be excited in the first 35 fs as the CC bond is elongated and the HCH angle opened after the passage through the twisted CI, which enhances the photoexcitation yield. Therefore, while relaxation through planar CI would be slower, the higher internal energy on D3 allows the twisted CI seam to come into play, enabling fast relaxation and keeping the delay with D0 on a 20 fs scale. The fact that a 25 fs delay has been observed also after excitation with higher photon energies^9^ suggests that the twisted CIs dominate the fast relaxation also when states higher than D3 are populated. Finally, Figure 4d–f shows clear oscillations, whose period is related to the twisting motion of the molecule and compares with the oscillations observed in Figure 3 for excitation with H11 and H13. Every time the molecular wavepacket revisits the CI seams responsible for relaxation, a fraction of it reaches the correct geometry and re-excitation by the IR pulse can happen. The oscillation amplitude appears to be strongly reduced in the experimental data while it is overestimated in the calculations. This can be due to the limitations of the SH simulation. It is worth noting that the oscillations in D1–D3 and D0 are almost out of phase. Therefore, if both the ground state and the excited states are populated, they are expected to partially cancel each other. This explains why no oscillations are observed in the C_2_H_4_^+^ yield obtained with H9 (Figure 3a).

In summary, we presented a detailed study of the ultrafast relaxation dynamics that follow the photoionization of a prototype organic molecule like ethylene. Few-femtosecond EUV pulses produced with a TDCM are used to create different initial coherent populations of the first four cationic states. The subsequent relaxation dynamics are interrogated in a pump–probe fashion by a 15 fs IR pulse which further excites the molecule and induces H and H_2_ loss. As a result, we observe a bleaching of the molecular cation signal, whose exact timing depends on the initially deposited energy. In particular, when the first excited states are populated, the bleaching peaks about 20 fs after ionization, while when the molecule is left in the cation ground state, H and H_2_ loss is a maximum when IR and EUV pulses overlap in time. Detailed comparison with the SH excited-state simulation allowed us to pinpoint the path followed by the molecule after excitation and demonstrate that the IR re-excitation proceeds through a resonant 3-photon absorption process between the cationic ground state and the third excited state. Moreover, we found the transition probability to correlate to the length of the CC bond and the aperture of the HCH angle. This allows us to prove that, while the ultrafast relaxation from the two lowest excited states is dominated by the planar CI, for higher excited states the twisted CI has a dominant role. Shedding new light on the ultrafast coupling between the internal degrees of freedom which mediate the ultrafast population transfer between the cationic states of a prototype molecule, our results are a step toward precise optical control of molecular reactions on ultrafast time scales.

## Supporting Information Available

Details on the experimental and computational methods, as well as extended experimental results (PDF)
