# Supplementary material for: Few-Femtosecond C2H4+ Internal Relaxation Dynamics Accessed by Selective Excitation
Source: J Phys Chem Lett. 2022 Nov 29;13(48):11169–75. doi: 10.1021/acs.jpclett.2c02763 (PMC9937561; doi:10.1021/acs.jpclett.2c02763)
Supplement: Supplementary file 1 — jz2c02763_si_001.pdf [file jz2c02763_si_001.pdf]

Few-femtosecond $C_2H_4^+$ Internal Relaxation Dynamics Accessed by Selective Excitation - Supporting Information

Matteo Lucchini,*,†,‡ Benoit Mignolet,¶ Mario Murari,†,‡ Cayo E. M. Gonçalves,¶ Giacinto D. Lucarelli,† Fabio Frassetto,§ Luca Poletto,§ Françoise Remacle,¶ and Mauro Nisoli†,‡

†*Department of Physics, Politecnico di Milano, 20133 Milano, Italy*

‡*Institute for Photonics and Nanotechnologies, IFN-CNR, 20133 Milano, Italy*

¶*Theoretical Physical Chemistry, UR MOLSYS, University of Liège, B4000 Liège, Belgium*

§*Institute for Photonics and Nanotechnologies, IFN-CNR, 35131 Padova, Italy*

E-mail: matteo.lucchini@polimi.it

Experimental Methods

15-fs IR pulses with central wavelength of ~ 811 nm and energy of 0.65 mJ are focused in a gas cell filled with Xe atoms. The comb of odd harmonics produced by high-order harmonic generation (HHG) is then sent into a time-delay compensated monochromator (TDCM), which is used to select a single harmonic. The TDCM consists of two sections working in a subtractive configuration to compensate for the temporal and spectral dispersion.[1] As a result, it is possible to select a single harmonic, while preserving its original temporal duration. Very recently we demonstrated the generation and characterization of an extreme-ultraviolet (EUV) pulse with duration down to 5 fs, limited only by the unavoidable residual geometrical dispersion of the TDCM.[2] In the present experiment we selected harmonics from the low energy part of the spectrum in order to excite mainly the ethylene cation and its first three excited states. In particular, we used the 9th, the 11th and the 13th harmonic.

Pulse characterization and delay-zero calibration

We estimated the time duration of each harmonic from a two-color photoemission experiment where the EUV radiation ionizes the molecule dressed by the IR femtosecond pulse. When the IR pulse overlaps in time with the EUV pulse, sideband signals appear in the photoelectron spectrum. By varying the delay between the IR and EUV it is possible to obtain a spectrogram (Fig. 1(a)) from which one can estimate the temporal properties of the light pulses.[3] In particular, when integrated in energy sideband signal lasts for a time duration τ_{SB} (full-width half-maximum, FWHM) equal to the cross-correlation between the IR pulse duration τ_{IR} and the harmonic duration τ_{EUV}: $\tau_{SB} = \sqrt{\tau_{IR}^2 + \tau_{EUV}^2}$.[3] To evaluate the harmonic time duration we estimated τ_{SB} by performing a Gaussian fit of the experimental data (Fig. 1(b)) and measuring τ_{IR} with a second-harmonic FROG experiment. We found that the time duration of the harmonic radiation ranges from 15 fs for H9 to 7 fs for the case of H13. The exact values are reported in Tab. 1.

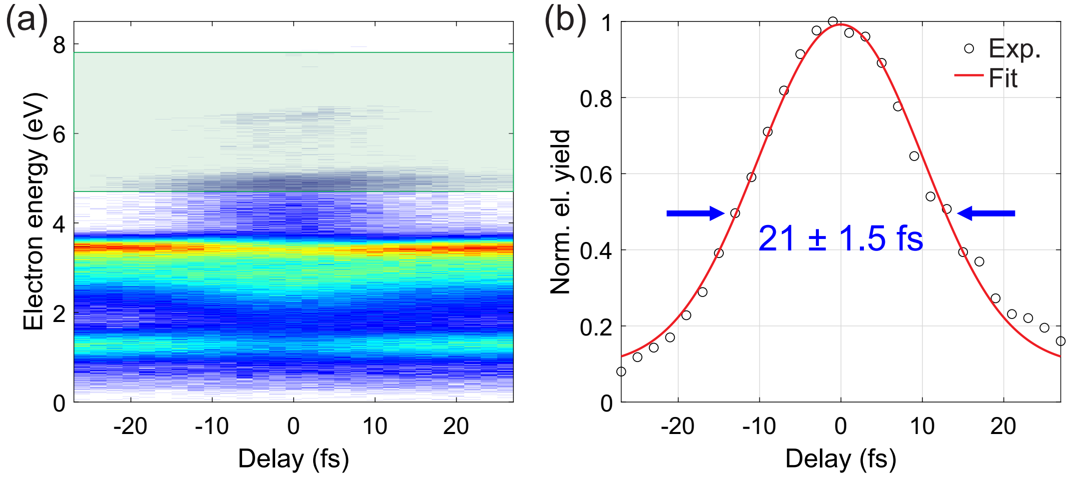

Figure 1: **(a)** Collection of photoelectron spectra ionized by H9 as a function of the delay with the IR pulse. **(b)** Black open circles, sideband electron yield obtained by energy integration over the green shaded area in (a), solid red curve, Gaussian fitting used to extract the cross-correlation time duration.

Table 1: Cross-correlation and harmonic duration extracted from the photoelectron pump-probe experiment for $\tau_{IR} = 15\,$fs.

HH	τ_{SB} (fs)	τ_{XUV} (fs)
9	21 ± 1.5	15 ± 2.2
11	18.5 ± 1.2	11 ± 1.8
13	17 ± 1.2	7.7 ± 2.4

As the SB yield peaks at the pump-probe overlap, the potoelectron spectrogram can be used to precisely locate the zero time delay τ_0. To this end, a couple of photoelectron experiments are performed immediately before and after each ion pump-probe measurement in order to calibrate the delay zero position and correct for possible linear drifts of the experimental interferometer.

Static ion yield

Figure 2(a) shows the main fragments produced by photoexcitation with the EUV pulses. As a consequence of the selective excitation, the fragment yields pertaining to different harmonics qualitatively differ from each other. Some of the observed differences can be understood by looking at the appearance potentials for the production of fragment ions as reported in Ref.[4] (Fig. 2(b)).

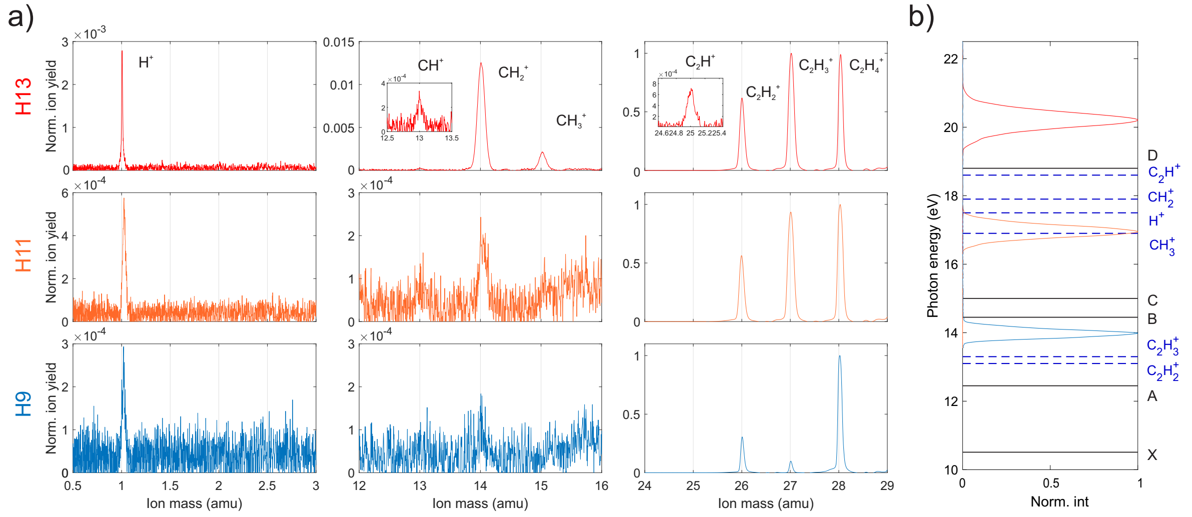

Figure 2: **(a)** Ion yield normalized for the maximum of the respective cation yield at 28 amu for photo-fragmentation by harmonic H13 (red curves, first row), H11 (orange curves, middle row), H9 (blue curves, bottom row). **(b)** Harmonic photon spectra together with the theoretical values of the appearance potentials for the production of fragment ions as reported in Ref.[4]

For example, the increasing production of H^+ fragment matches with the associated appearance potential which lies just above H11 at 17.5 eV (compare the plots in the first

column in Fig. 2(a)). The same behaviour is observed for the CH_2^+ and C_2H^+ fragments with threshold lying between H11 and H13 at 17.9 and 18.6 eV, respectively. The behaviour of the $C_2H_3^+$ fragment is instead not trivial. All three harmonics are supposed to have enough energy to create this fragment (appearance potential 13.3 eV), but the peak at 27 amu strongly increases while using H11 and H13. This observation suggests the existence of an "efficient" fragmentation path leading to $C_2H_3^+$ production which opens with excitation energy between 14 and 17 eV. As discussed in the main text, this phenomenon is related to the internal energy redistribution durign the non-adiabatic relaxation processes.

Pump-probe results

In the following we report the results obtained by using an IR intensity of $I_{IR} = 3.34 \times 10^{12}\,\mathrm{W/cm^2}$ and averaging over several separate scans. We plot the differential fragment yield, $\Delta Y(\tau)$, defined as:

$$\Delta Y(\tau) = \frac{Y_{IR}(\tau) - Y_0(\tau)}{Y_0(\tau)}, \tag{1}$$

where τ is the pump-probe delay, Y_{IR} is the ion yield measured when the IR field is turned on and Y_0 is the ion yield measured when the IR field is turned off. In all figures, dots represent the average of the scans while the error bars cover twice their standard deviation. Error propagation has been taken into account while calculating the standard error bars. Solid lines show the fitting result.

Harmonic 13

Figure 3 reports the relative ion yield of the fragments that exhibit an appreciable pump-probe signal after H13 excitation. All the features observed qualitatively agree with the previous measurements based on attosecond pulse trains.[5]

The cation signal (Fig. 3 top left panel) shows a delayed bleaching centred at about 23 fs

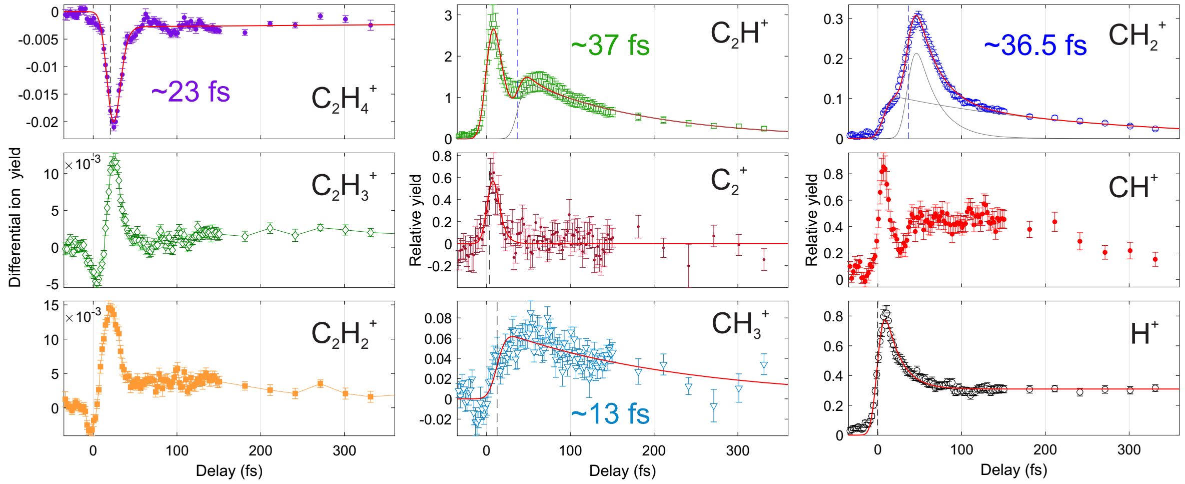

Figure 3: Relative ion yield as a function of pump-probe delay after H13 ionization. Dots represents the experimental data. Continuous red lines are the fitting results. The labels and the vertical dashed line report the time at which the EMG fitting curves are centred.

after the pump-probe temporal overlap. The origin of this signal is widely described in the main text. The differential yields of ${C_2H_3}^+$ and ${C_2H_2}^+$ display a derivative behaviour which seems to be strongly correlated to the cation bleaching signal (compare the respective panels in Fig. 3). Both fragment yields can be fitted with two exponentially modified gaussians (EMGs), one negative and centered around $\tau = 0$ fs and the other positive and delayed in time. The extracted center time for the positive EMGs fairly agrees with the 20 fs delay observed in the cation bleaching. It is important to notice that an accurate extraction of the fitting parameters is very challenging for these fragments as different combinations of positive and negative EMGs would lead to the same final curve. The differential yields of C_2H^+ and ${CH_2}^+$ can be fitted with two positive EMGs, one centered at $\tau = 0$ fs and the other delayed by roughly 40 fs. A similar qualitative behaviour is observed for CH^+. In case of C_2H^+ the fit suggest a fast decay rate of $T_d^{(1)} = 13.7$ fs for the EMG component centered around $\tau = 0$ fs and a slower decay rate of $T_d^{(2)} = 151.8$ fs for the delayed EMG centered at 37.2 fs (see the solid gray lines in the figure panel). For ${CH_2}^+$, instead, the fit parameters are $T_d^{(1)} = 234.3$ fs for the EMG component centered around $\tau = 0$ fs and $T_d^{(2)} = 25.2$ fs for the delayed EMG centered at 36.5 fs. The ${CH_3}^+$ fragment shows a delayed peak which appears

around 12.6 fs, considerably shorter than the 30 fs observed with higher excitation energies.[5] Finally, the H^+ signal (Fig. 3 bottom right panel) can be fitted with a positive decay and a formation. Both are centered around 0 fs pump-probe delay. The decay time extracted from the fit is $T_d = 18.2$ fs,suggesting an almost instantaneous response. As we expect the process of hydrogen loss to be the easiest to trigger by the IR pump, the increasing proton signal around zero time pump-probe delay proves the reliability of our pump-probe delay axis calibration.

Harmonic 11

Excitation with harmonic 11 (Fig. 4) gives similar results to the ones obtained with H13. The cation signal (Fig. 4 top left panel) shows the same belated bleaching behaviour with weak oscillations at bigger delays. As in the previous case, ${C_2H_3}^+$ is characterized by a derivative behaviour where the negative contribution seems to be correlated to the cation bleaching as in Fig. 3.

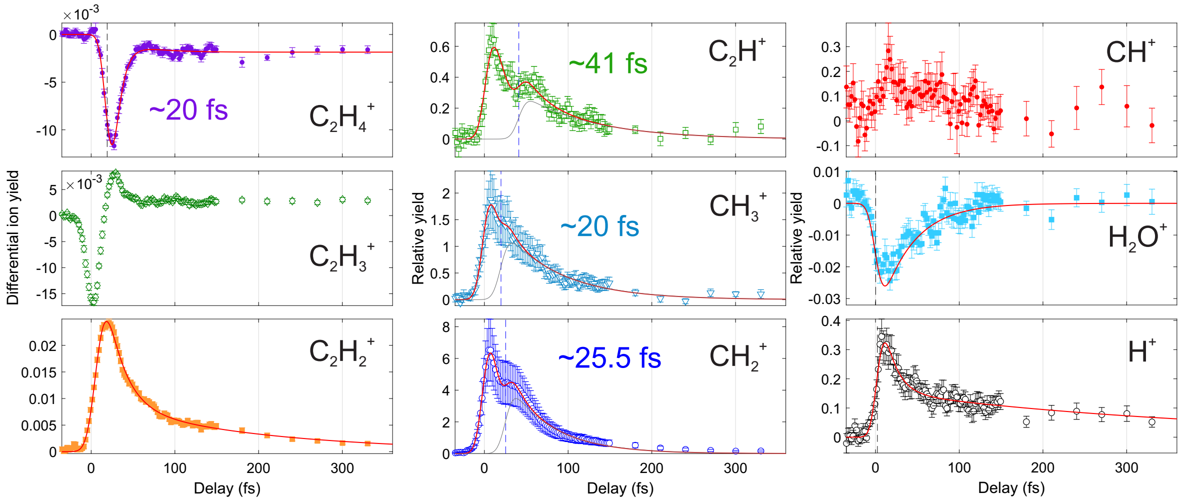

Figure 4: Differential ion yield as a function of pump-probe delay after H11 ionization. Dots represents the experimental data. Continuous red lines are the fitting result. The labels and the vertical dashed lines report the time at which the EMG fitting components are centred.

The fragment ${C_2H_2}^+$ has instead a different behaviour, changing from a derivative signal for H13 to a double positive decay centered around the pump-probe delay zero. The fastest

decay has a constant of $T_d^{(1)} = 21.6\,\text{fs}$ while the slower component has a decay constant of $T_d^{(2)} = 188\,\text{fs}$.

C_2H^+, $CH_3{}^+$ and $CH_2{}^+$ can be fitted with two EMGs, one centered around delay zero and one delayed. In all the cases, the EMG centered at 0 fs has a very fast decay, suggesting an almost instantaneous signal that follows the cross-correlation of the pulses. The delayed contribution is centered between 25 and 40 fs after delay zero and has a decay constant of $T_d^{(2)}$ of the order of 50 to 80 fs.

Figure 4, bottom right panel, shows the H^+ signal which can be fitted with a double exponential decay with an instantaneous contribution and a slower EMG, $T_d = 388\,\text{fs}$, centered at 0 fs. Again this proves that our pump-probe delay calibration is reliable. A further prove is to be found in the water signal from the chamber background. Indeed, the H_2O^+ differential yield (middle right panel) is characterized by a bleaching fitted by one EMG with decay rate of $T_d = 41\,\text{fs}$ and well centered around 0 fs. The OH^+ signal (not shown), exhibits a mirror dynamical behavior, well described by a positive EMG centered at zero.

Harmonic 9

When the 9th harmonic is used to prepare the cation, appreciable dynamics are found only in the three heaviest fragments (Fig. 5). Weaker dynamics can be seen in the fragments $CH_3{}^+$ and $CH_2{}^+$. The cation signal (Fig 5 top left panel) is bleached around the zero time delay. No clear oscillation appears in the tail at positive delays.

The deprotonated cation, $C_2H_3{}^+$, does no longer evolve with a derivative profile, but shows a clear double decay behaviour (see Fig. 5 second row, first column) which can be fitted with two EMGs, centered at pump-probe overlap and described by the time constants $T_d^{(1)} = 51\,\text{fs}$ and $T_d^{(2)} = 309\,\text{fs}$. On the contrary, $C_2H_2{}^+$ (Fig. 5 third row, first column) follows a derivative profile as in case of H13 excitation. Moreover, in this case we observe a clear slow decay, which is not found in the H13 data (compare with Fig. 3 first column,

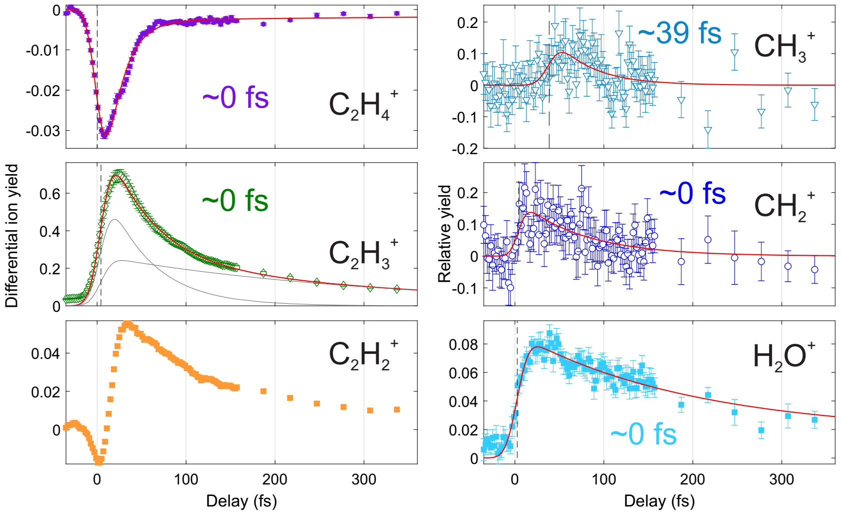

Figure 5: Differential ion yield as a function of pump-probe delay after H9 ionization. Dots represents the experimental data. Continuous red lines are the fitting result. The labels and vertical dashed lines report the time at which the EMG fitting curves are centred.

third row).

The water signal reported in Fig. 5 serves as a double check for our delay zero calibration. No appreciable signal is observed in the H^+ fragment.

IR intensity dependence

We studied the IR intensity dependence of the differential ion yields for the three chosen harmonics to test the robustness of the results. Figure 6 reports the behaviour of three representative ions at four IR intensities, $I_{IR} = 4.65 \times 10^{11}$, 1.29×10^{12}, 2.19×10^{12} and $3.34 \times 10^{12}\,\mathrm{W/cm^2}$ (blue, green, yellow and red colors, respectively). As one can notice, the pump-probe signals exhibit the same qualitative behaviour in the IR intensity range under investigation, thus underling the robustness of the results.

Fitting model

To extract the ion yield variation induced by the pump IR pulse, we integrate each ion peak obtained with the time-of-flight spectrometer along the amu axis over a window of

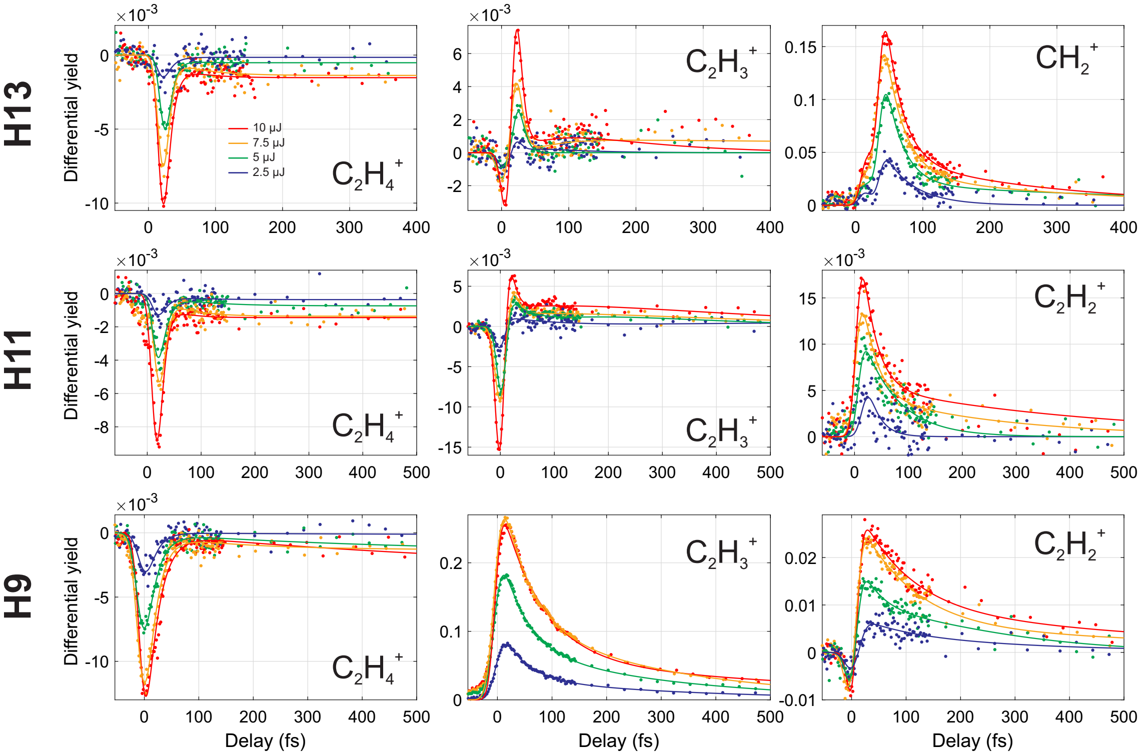

Figure 6: Relative ion yields as a function of pump-probe delay and for different IR intensities. First row: ionization with H13. Second row: ionization with H11. Third row: ionization with H9. The selected ions are labelled in each panel. Dots represent the experimental data, solid lines the resulting fit with a sum of exponentially modified Gaussians (EMGs). Red, yellow, green and blue indicate a pulse energy of 10, 7.5, 5 and 2.5 μJ, corresponding to an IR intensity of 3.34×10^{12}, 2.19×10^{12}, 1.29×10^{12} and 4.65×10^{11} W/cm^2, respectively.

fix width of 0.2 amu and get the delay dependent quantity $Y_i^{IR}(\tau)$. We then perform the same integration in the data without the pump, Y_i^0, and construct the differential yield as described in the previous section. For each ion, the differential yields are fitted with a sum of EMGs of the form:

$$Q(t) = \sum_{i=1}^{n} A_i D_i(t) + \sum_{j=1}^{N} B_j F_j(t) \tag{2}$$

where the A_i and B_j are the amplitude of the EMG contributions representing a decay, D_i, or a formation, F_i. In case of a decaying contribution, the function D_i is given by the convolution of a normalized Gaussian instrumental response function, $g(t)$, with an exponential decay, $f(t)$:

$$D(t_i, \sigma_i, \alpha_i, t) = g(t) * f(t) = \frac{\alpha_i}{2} \exp\left\{\frac{\alpha_i}{2}\left(2t_i + \alpha_i\sigma^2 - 2t\right)\right\} \operatorname{erfc}\left\{\frac{t_i + \alpha_i\sigma^2 - t}{\sqrt{2}\sigma}\right\} \tag{3}$$

where

$$g(t) = \frac{1}{\sigma\sqrt{2\pi}} e^{-\frac{(t-t_i)^2}{2\sigma^2}} \tag{4}$$

$$f(t) = \alpha_i e^{-\alpha_i t} H(t) \tag{5}$$

With $H(t)$ being the Heaviside function. The decay rate of the exponential $T_d^{(i)}$ is given by $1/\alpha_i$.

In the case of a formation, the convolution with the instrumental response function gives a term of the kind:

$$F(t_i, \sigma_i, \alpha_i, t) = \frac{\alpha_i}{2}\left[\operatorname{erfc}\left\{\frac{t_i - t}{\sqrt{2}\sigma}\right\} - \exp\left\{\frac{\alpha_i}{2}\left(2t_i + \alpha_i\sigma^2 - 2t\right)\right\} \operatorname{erfc}\left\{\frac{t_i + \alpha_i\sigma^2 - t}{\sqrt{2}\sigma}\right\}\right] \tag{6}$$

While fitting a particular ion yield with the function $Q(t)$, we used the minimum number of formations and decays, capable to qualitatively follow the data. The quantities α_i, t_i and the amplitudes A_i (B_i) are free parameters in the procedure while the width of the

instrumental response function, σ, is fixed to the value obtained with the photoelectron cross-correlation measurements (Tab. 1) for all the contributions.

To fit the oscillations observed in the differential $C_2H_4^+$ yield after H11 and H13 ionization, a sinusoidal term is added to the fitting function so that the function D_i becomes:

$$D(t_i, \sigma_i, \alpha_i, t) = \frac{\alpha_i}{2} \exp\left\{\frac{\alpha_i}{2}\left(2t_i + \alpha_i\sigma^2 - 2t\right)\right\} \operatorname{erfc}\left\{\frac{t_i + \alpha_i\sigma^2 - t}{\sqrt{2}\sigma}\right\} \left[1 + a_i \cos\left(b_i t + c_i\right)\right] \tag{7}$$

The amplitude, a, the frequency b and the phase c are new free parameters of the fitting procedure.

Computational method

The molecular dynamics and nonradiative relaxation resulting from the photoionization of the neutral ethylene molecule to the cationic electronic states D0, D1, D2 and D3, have been investigated using surface hopping (SH) with the SHARC (Surface Hopping including Arbitrary Couplings) software[6] and Born-Oppenheimer molecular dynamics.[7] For each cationic state, 300 trajectories are run where the initial conditions have been sampled from a Wigner distribution of the neutral ground state. The electronic structure and nonradiative couplings are computed at the SA(7)-CASSCF(9MO,11 electrons) with the 6-311G(d,p) basis set. Once the wavepacket has relaxed on the ground state for more than 10 fs, the surface hopping SHARC dynamics is switched to the BOMD (Born-Oppenheimer molecular dynamics)[8] as implemented in Gaussian16.[9] The surface hopping dynamics is based on state average (SA) CAS electronic structure,[10] which is well suited for computing nonradiative couplings close to the Franck-Condon region, but not for describing the fragmentation due to the limited number of orbitals included in the active space in the SA-CAS electronic structure. Therefore the dynamics is switched to BOMD at the DFT B3LYP/6-31G(d,p) level to compute the dynamics up to 2 ps after the nonradiative relaxation. We therefore make the assumption that once the wavepacket has relaxed on the ground state there is no

nonradiative transitions to excited states. The dissociation must then occur on the ground state (i.e. not mediated by excited states), which is usually the main dissociation channel. Slow dissociation processes occurring on the picosecond time scale are not included in the dissociation yield of Table 1 in the main manuscript due to the 2 ps time window of the simulations.

The non-radiative relaxation of the cationic states D1, D2 and D3 is reported in Fig. 7. The excited state lifetime (i.e. the time it takes for half of the wavepacket to relax on D0) is respectively 12.6 fs, 27.1 fs and 36.6 fs for the dynamics starting from D1, D2 and D3. The Fig. 3d-f in the main paper represent the nonradiative relaxation corresponding to a weighted average of the dynamics initiated on the states D0 to D3 where the weight of the initial states depends on the initial population excited by each harmonic (see Fig. 1d of the main text).

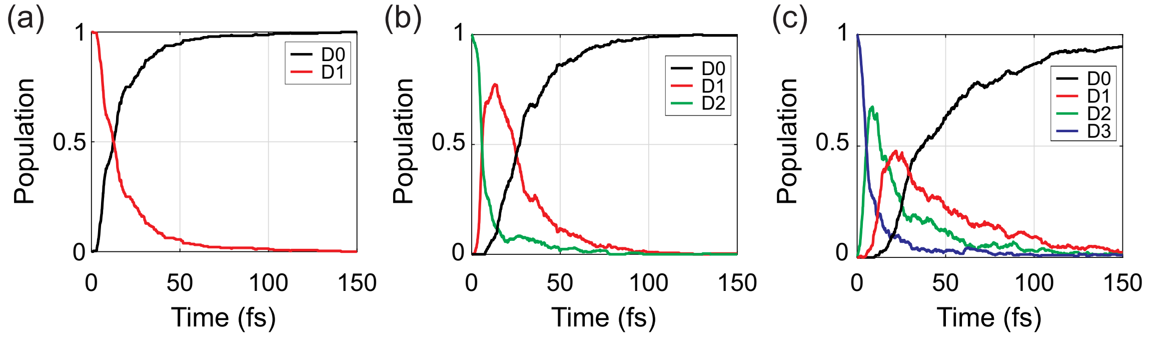

Figure 7: Nonradiative relaxation from the D1 **(a)**, D2 **(b)** and D3 **(c)** state.

For the dynamics starting from the cationic state D0, the excited states SH simulations based on SA-CASSCF electronic structure have been carried out until 100 fs after excitation. Therefore, the mean energy gap between the ground and the excited states of the 300 SH simulations as well as the associated transition dipole moment can be reported up to 100 fs as in Fig. 4a-b of the main manuscript. If the energy between D0 and the excited states D1 to D3 is within ±0.1 eV of an integer number of the photon energy, then we consider the transition resonant. Averaging over the 300 simulations gives the percentage of the wavepacket being

resonant in time (Fig. 4(a) of the main text). In addition, the photoexcitation will be favored by a large transition dipole (Fig. 4(b) of the main text), which is correlated to an elongation of the CC bond and an opening of the HCH angle. The average norm of the transition dipole for the 300 SH simulations in a 100-fs time window on D0 is reported as a function of the CC bond and HCH angle in Fig. 4b of the main manuscript. The white areas in the same figure represent zones of the potential energy surface that have not be explored by the WP on the ground state. For the dynamics starting on the excited states D1 to D3, the photoexcitation probability reported in Figs. 4d-e of the main text is derived from the geometry of the molecule for trajectories that have relaxed on D0 as the dynamics is switched from SH to BOMD once on D0. Therefore, the transition dipole moment and energy gap are not available.

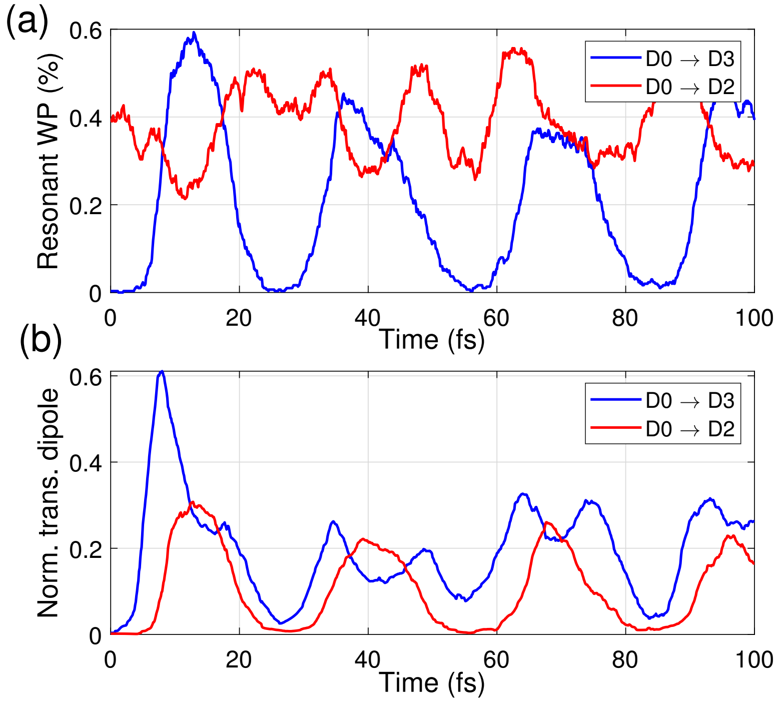

Figure 8: **(a)** Fraction of trajectories that are 3-photon resonant between D0 and D2 or D3. The fraction of the trajectories being 2-photon resonant D0-D2 or D0-D3 is always below 3%. **(b)** Time evolution of the mean of the norm of the transition dipole moment between D0-D3 and D0-D2.

In the main text the correlation between the CC bond elongation and the HCH angle

opening with the 3-photon photoexcitation probability of the D0 to D3 transition is emphasized as it provides a reasonable explanation for the increase of the H and H_2 fragments for delayed time of 20 fs between the two pulses. Others electronic transitions can also be induced by the IR pulse by multiphoton transitions such as the D0→D2 transition. However, at short delay time (< 10 fs) 2/3 of the wavepacket on D0 is 3-photon resonant D0-D3 while only 1/3 is 3-photon resonant D0/D2 ((Fig. 8(a))). 2-photon transitions from D0-D2 or D0-D3 are negligible. Furthermore, the transition dipole moment is about twice lower for the D0/D2 transition in the early stage of the dynamics (Fig. 8(b)). This suggests that the sharp increase of dissociation yield observed for the H9 harmonic is mainly due to the D0-D3 transitions. The D0-D2 transition only seems to be correlated to the CC bond elongation while for the D0-D3 transition is correlated to both the CC bond elongation and to the HCH angle opening, which strongly increases the photoexcitation probability.

High excitation region

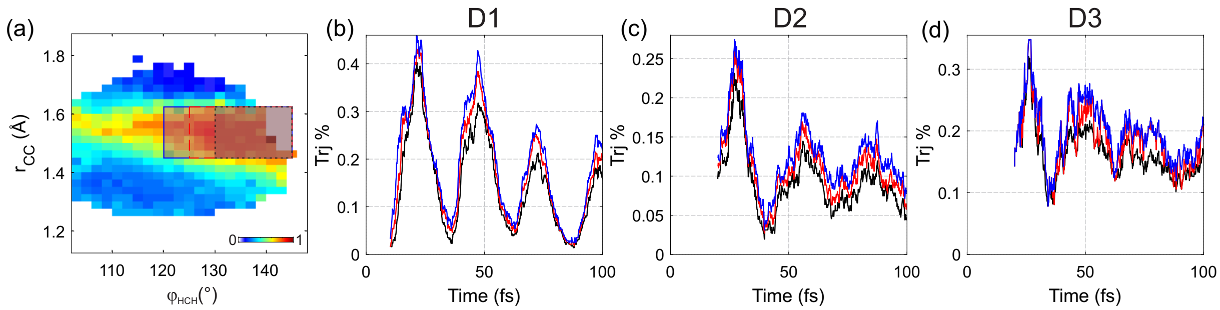

Figure 9: **(a)** D0-D3 transition dipole probability as a function of r_{CC} and φ_{HCH}. The black rectangle delimits the region 1.45 Å$\leq r_{\mathrm{CC}} \leq$ 1.625 Å and $130° \leq \varphi_{\mathrm{HCH}} \leq 145°$. The red and blue rectangle describe a region with increased angular window of $125° \leq \varphi_{\mathrm{HCH}} \leq 145°$ and $120° \leq \varphi_{\mathrm{HCH}} \leq 145°$, respectively. **(b)** Percentage of trajectories falling in the black, red or blue rectangle (same color code), for initial excitation on D1. **(c)**, **(d)**, same quantities but for initial excitation on D2 and D3, respectively.

To identify the relaxation dynamics probed by the 3-photon IR re-excitation mechanisms, we defined a region of high excitation as in the gray box of Fig. 4(c) of the main manuscript

and in Fig. 9(a) (1.45 Å$\leq$ r_{CC} $\leq$ 1.625 Å and 130° $\leq$ φ_{HCH} $\leq$ 145°). This assures high photoexcitation probability from D0 to D3 is high. It is worth noticing that a decrease of the lower limit of the angle would slightly shift upward the fraction of resonant trajectories, without any impact on the interpretation of the results. To make this point clear, we recalculated the percentage of trajectories following into the selected region by reducing the minimum angle to 125° and 120°. The results are reported in Figs. 9(b)-(d). Black indicates the data reported in the main manuscript, red the region 125° $\leq$ φ_{HCH} $\leq$ 145°, blue the region 120° $\leq$ φ_{HCH} $\leq$ 145°.

Finally, we have checked the robustness of the physical picture presented in Figs. 4(d)-(f) of the main manuscript against the chosen definition of the high-excitation region. Figure 10 compares the results presented in the main text (solid colored curves) with the calculations performed by shifting the original box 5° towards lower angular values, i.e. 125° $\leq$ φ_{HCH} $\leq$ 140° (grey-dashed curves). We found that the total fraction of relevant trajectories (first column in Fig. 10) is not strongly affected as well as the planar vs. twisted ratio (second and third column in Fig. 10).

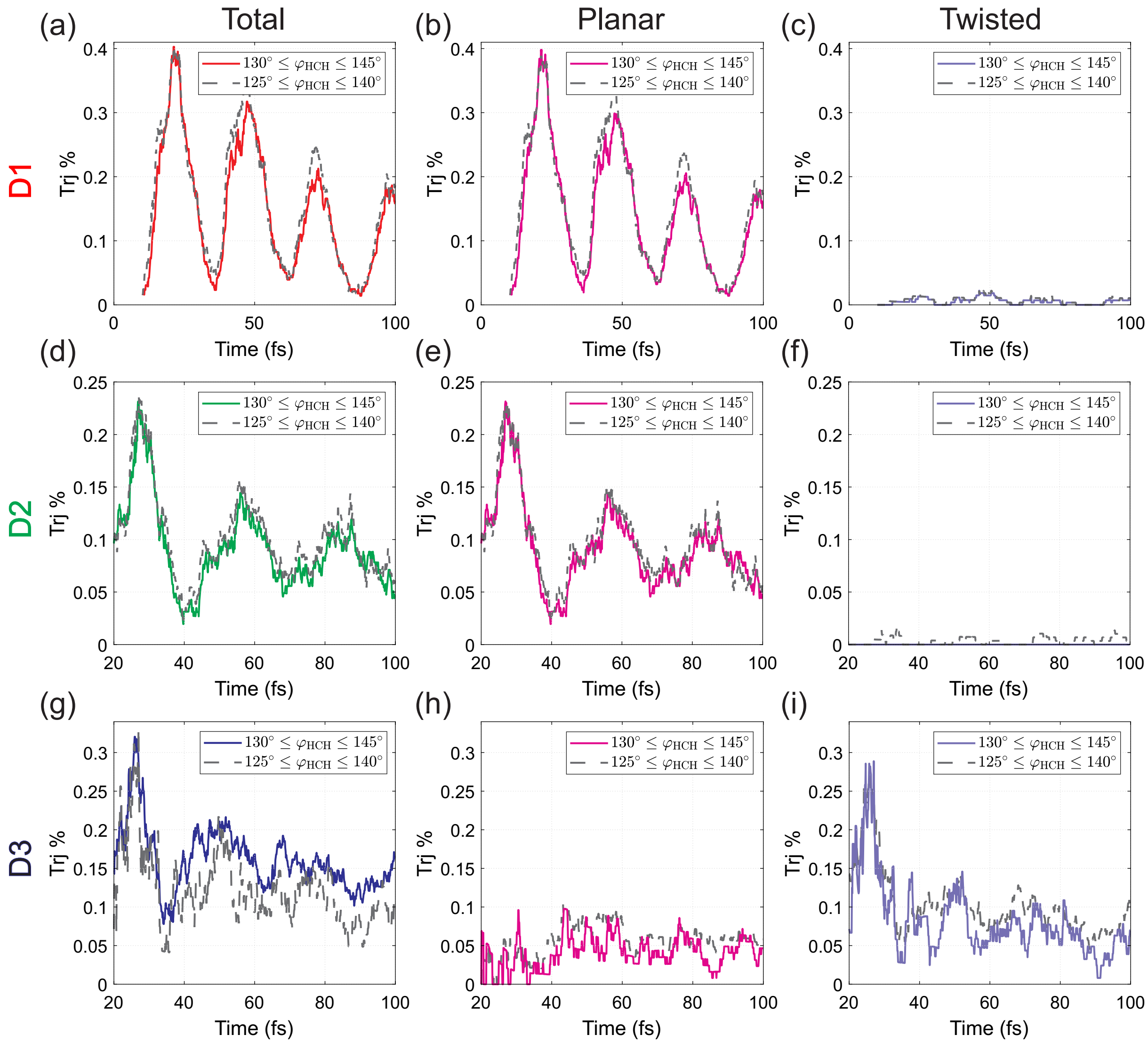

Figure 10: Percentage of trajectories falling into the region defined by $1.45\,\text{Å} \leq r_{\text{CC}} \leq 1.625\,\text{Å}$ and $130^\circ \leq \varphi_{\text{HCH}} \leq 145^\circ$ (solid colored curves) for initial excitation on D1, first row, D2, second row and D3, third row. The first column presents the total trajectories falling in the selected region, the second column its fraction which has relaxed through a planar D0/D1 CI while the third column the fraction that as relaxed through a twisted D0/D1 CI. In all panels, the dashed grey curves represent the same quantities but for a highlighted region shifted by 5° downwards, i.e. defined by $1.45\,\text{Å} \leq r_{\text{CC}} \leq 1.625\,\text{Å}$ and $125^\circ \leq \varphi_{\text{HCH}} \leq 140^\circ$.

References

(1) Poletto, L.; Villoresi, P.; Frassetto, F.; Calegari, F.; Ferrari, F.; Lucchini, M.; Sansone, G.; Nisoli, M. Time-delay compensated monochromator for the spectral selection of extreme-ultraviolet high-order laser harmonics. *Review of Scientific Instruments* **2009**, *80*, 123109

.

(2) Lucchini, M.; Lucarelli, G. D.; Murari, M.; Trabattoni, A.; Fabris, N.; Frassetto, F.; Silvestri, S. D.; Poletto, L.; Nisoli, M. Few-femtosecond extreme-ultraviolet pulses fully reconstructed by a ptychographic technique. *Opt. Express* **2018**, *26*, 6771–6784

.

(3) Moio, B.; Dolso, G. L.; Inzani, G.; Di Palo, N.; Borrego-Varillas, R.; Nisoli, M.; Lucchini, M. Time-frequency mapping of two-colour photoemission driven by harmonic radiation. *Journal of Physics B: Atomic, Molecular and Optical Physics* **2021**, *54*, 154003

.

(4) Mackie, R. A.; Scully, S. W. J.; Sands, A. M.; Browning, R.; Dunn, K. F.; Latimer, C. J. A photoionization mass spectrometric study of acetylene and ethylene in the VUV spectral region. *Int. J. Mass Spectrom.* **2003**, *223-224*, 67–79

.

(5) Ludwig, A.; Liberatore, E.; Herrmann, J.; Kasmi, L.; López-Tarifa, P.; Gallmann, L.; Rothlisberger, U.; Keller, U.; Lucchini, M. Ultrafast Relaxation Dynamics of the Ethylene Cation $C_2H_4^+$. *The Journal of Physical Chemistry Letters* **2016**, *7*, 1901–1906, PMID: 27139223

.

(6) Richter, M.; Marquetand, P.; González-Vázquez, J.; Sola, I.; González, L. SHARC:

ab Initio Molecular Dynamics with Surface Hopping in the Adiabatic Representation Including Arbitrary Couplings. *Journal of Chemical Theory and Computation* **2011**, *7*, 1253–1258

.

(7) Millam, J. M.; Bakken, V.; Chen, W.; Hase, W. L.; Schlegel, H. B. *Ab initio* classical trajectories on the Born?Oppenheimer surface: Hessian-based integrators using fifth-order polynomial and rational function fits. *The Journal of Chemical Physics* **1999**, *111*, 3800–3805

.

(8) Helgaker, T.; Uggerud, E.; Jensen, H. J. A. Integration of the classical equations of motion on ab initio molecular potential energy surfaces using gradients and Hessians: application to translational energy release upon fragmentation. *Chemical Physics Letters* **1990**, *173*, 145–150

.

(9) Frisch, M. J.; Trucks, G. W.; Schlegel, H. B.; Scuseria, G. E.; Robb, M. A.; Cheeseman, J. R.; Scalmani, G.; Barone, V.; Petersson, G. A.; Nakatsuji, H. et al. Gaussian?16 Revision C.01. 2016; Gaussian Inc. Wallingford CT

.

(10) Werner, H.; Knowles, P. J. A second order multiconfiguration SCF procedure with optimum convergence. *The Journal of Chemical Physics* **1985**, *82*, 5053–5063

.
